# The Development of IgE Multiple Myeloma Following Treatment for Locally Advanced Prostate Cancer

**DOI:** 10.7759/cureus.59732

**Published:** 2024-05-06

**Authors:** Motoki Fujita, Issei Suzuki, Tomoya Mizuno, Hiroyuki Kobayashi, Tsunehito Kambara

**Affiliations:** 1 Urology, Nasu Red Cross Hospital, Otawara, JPN; 2 Hematology, Nasu Red Cross Hospital, Otawara, JPN

**Keywords:** psa, multiple myeloma, acute renal injury, ige multiple myeloma, prostate cancer

## Abstract

This case report documents the diagnosis of multiple myeloma (MM) in a 74-year-old man following treatment for locally advanced prostate cancer. It is important to include MM in the differential diagnosis when the patient presents with nonspecific symptoms such as back pain, anemia, and renal impairment in the absence of a prominent increase in prostate-specific antigen (PSA). The present case was diagnosed as IgE MM with a poor prognosis. Prompt diagnosis and intervention of MM is necessary to avoid complications, including renal impairment.

## Introduction

Multiple myeloma (MM) is the second most common hematologic malignancy after non-Hodgkin's lymphoma in elderly men [1]. Prostate cancer is likewise the second most diagnosed cancer in men worldwide, and older age has been identified as a risk factor [2]. Characteristically, both malignancies present with bone involvement, and although rare, simultaneous diagnosis of prostate cancer and MM has been reported [3]. IgE MM is particularly rare, accounting for only 0.1% of cases, and has a poor prognosis [4]. The presence of renal involvement in MM is a poor prognostic factor [5]. The present case details a case of MM diagnosed after treatment for prostate cancer with symptoms of back pain, anemia, and renal dysfunction.

## Case presentation

A 74-year-old man presented to the emergency department of our hospital complaining of back pain. He also recently complained of shortness of breath and fever. He reported that these symptoms worsened as his physical activity increased. He had no symptoms of nausea, vomiting, or bloody stool, and no urinary symptoms such as hematuria or pain during urination. He had percussion tenderness in his right lumbar region but no induration or pain in his epididymis and no tenderness in Douglas fossa on rectal examination.

His medical history included prostate cancer, for which he had undergone radical surgery six months ago. He was diagnosed with localized prostate cancer (clinical T2aN0M0) by high prostate-specific antigen (PSA) level (PSA 4.14 ng/mL), magnetic resonance imaging (MRI), transperineal prostate biopsy, and computed tomography (CT) scans. He underwent a robot-assisted radical prostatectomy (RARP). The pathological examination revealed an elevation of the stage from localized cancer to locally advanced cancer. TNM stage, post-RARP was pathological T3aN0M0, with a Gleason score of 3+4=7.

He had no regular medications. Laboratory findings highlighted an elevated inflammatory response, with increased C-reactive protein (CRP) levels and elevated white blood cell counts (Tables 1, 2).

**Table 1:** Blood tests at the time of consultation LDH: lactate dehydrogenase; Cre: creatinine; BUN: blood urea nitrogen; WBC: white blood cell; RBC: red blood cell; MCV: mean corpuscular volume; MCH: mean corpuscular hemoglobin; MCHC: mean corpuscular hemoglobin concentration

Blood analysis and count		
Parameters	Patient values	Reference range
LDH (U/L)	158	124-222
Sodium (mmol/L)	136	138-145
Potassium (mmol/L)	4.4	3.6-4.8
Chloride (mmol/L)	107	101-108
Calcium (mg/dL)	8.5	8.8-10.1
Phosphorus (mg/dL)	3.7	2.7-4.6
Cre (mg/dL)	4.5	0.65-1.07
BUN (mg/dL)	43.4	8-20
WBC (103/uL)	10.3	3.3-8.6
RBC (106/uL)	2.28	4.35-5.55
Hemoglobin (g/dL)	7.6	13.7-16.8
Hematocrit (%)	22.4	40.7-50.1
MCV (fL)	98.2	83.6-98.2
MCH (pg)	33.3	27.5-33.2
MCHC (g/dL)	33.9	31.7-35.3
Platelet (103/uL)	224	158-348

**Table 2:** Urine tests at the time of consultation WBC (/HPF): white blood cells per high-power field; RBC (/HPF): red blood cells per high-power field

Urine analysis		
Parameters	Patient values	Reference range
Specific gravity	1.012	1.005-1.022
pH	5.5	4.6-7.5
WBC (/HPF)	>100	-
RBC (/HPF)	0-1	-
Protein	2+	-

Additionally, the results indicated anemia and acute renal impairment, with urinalysis revealing pyuria. CT scans showed no findings suggestive of pneumonia, and no findings such as renal enlargement, thickening of Gerota's fascia, thickening of the bridging septum, or increased concentration of fat tissue in the perinephric space suggestive of pyelonephritis. Considering his clinical symptoms, he was diagnosed with acute pyelonephritis and started antibiotics. His fever subsided after a few days, but his back pain did not improve. Laboratory findings revealed worsening of his anemia and renal dysfunction.

**Figure 1:**
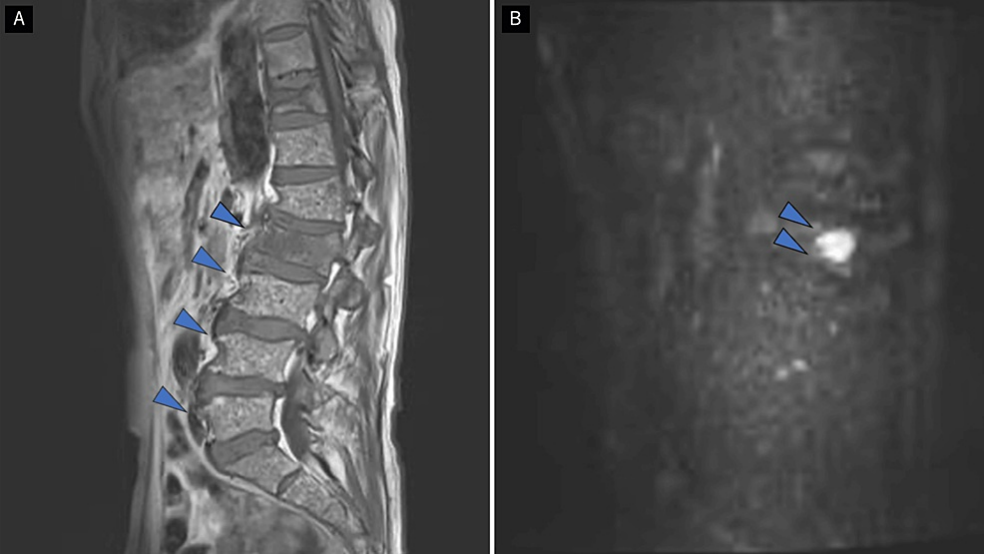
Spine MR imaging (A) T1-weighted MR imaging revealed a "salt-and-pepper pattern" characterized by numerous small foci of low signal intensity (indicated by blue arrows). (B) On high-b value DWI images from Th12 to L2, abnormal high signal intensities were observed in the bone marrow, with a notably strong abnormal signal confirmed at L2 (indicated by blue arrows). DWI: diffusion-weighted imaging

MRI to search for the cause of his back pain revealed a compression fracture of the second lumbar vertebra and a high-signal area from the 12th thoracic vertebra to the fifth lumbar vertebra, raising suspicion of bone metastases from prostate cancer or bone lesions from MM (Figure 1).

Although the PSA level was below sensitivity, the markers progastrin-releasing peptide (proGRP) and neuron-specific enolase (NSE) were measured and were within reference values due to concerns about neuroendocrine differentiation (NED) of prostate cancer. He consulted a hematologist for further investigation of the bone involvement of MM. Further diagnostic tests were conducted, including additional urine and serum analyses. Urine tests revealed the presence of Bence-Jones protein kappa (BJP-κ) type. Serum analyses detected an M protein component, specifically IgE, with a concentration exceeding 16,000 mg/L. A serum immunoglobulin free light chain (FLC) assay indicated a significant increase in kappa light chains (3,620 mg/L) relative to lambda light chains (11.7 mg/L), with a markedly elevated kappa/lambda ratio of 309.32 (Table 3).

**Table 3:** Additional blood tests ProGRP: progastrin-releasing peptide; NSE: neuron-specific enolase; β-2MG: beta-2 microglobulin

Additional blood tests		
Parameters	Patient values	Reference range
ProGRP (pg/ml)	72.3	0-81
NSE (ng/ml)	8.7	0-10
IgG (mg/dL)	395	861-1747
IgA (mg/dL)	18	93-393
IgM (mg/dL)	10	33-183
IgE (mg/dL)	>16000	0-173
Free light chain kappa (mg/L)	3620	3.3-19.4
Free light chain lambda (mg/L)	11.7	5.7-26.3
kappa/lambda	309.32	0.26-1.65
β-2MG (mg/L)	15.8	1.0-1.9

**Figure 2:**
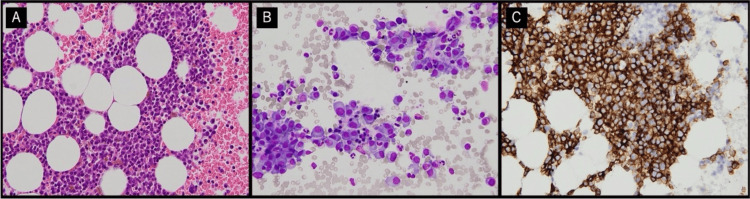
Microscopic findings of plasma cells in a frozen section of a bone marrow aspiration (A) Hematoxylin and eosin staining (400×), (B) May-Giemsa staining (400×), (C) CD138 immunostaining (400×).

Following the bone marrow aspiration and subsequent diagnosis of MM, characterized by a strong positivity for the CD138 cell surface marker, the patient commenced anticancer chemotherapy comprising daratumumab, bortezomib, and dexamethasone within the hematology department (Figure 2).

After initiation of treatment for MM, the patient's back pain improved markedly. Furthermore, six months after the start of treatment, there was a marked improvement in anemia and renal function, with no further deterioration.

## Discussion

In metastatic prostate cancer, PSA levels may not rise or may rise only slightly, even when symptoms worsen or imaging studies show rapid disease progression. In such cases, NED should be considered [6]. NSE, chromogranin A (CgA), and proGRP are representative markers of neuroendocrine prostate cancer. These can be detected immunohistochemically in biopsy specimens or by measuring their levels in peripheral blood to assess NED and have been reported to be useful in predicting prostate cancer prognosis [7]. Mahdy et al. also reported a case of bone metastasis without a significant increase in PSA several years after treatment for localized prostate cancer [8]. Therefore, the possibility of bone metastasis from NED prostate cancer should be considered when new bone lesions appear without an elevated PSA.

On the other hand, elderly patients with prostate cancer naturally belong to the high-risk group for other organ cancers. Therefore, even during prostate cancer treatment, it is essential to always be vigilant for the presence of concurrent malignancies.

MM is another disease that rapidly increases in incidence after the age of 60 years, and since it coincides with the age group in which prostate cancer occurs most frequently, it should be one of the differential diagnoses in prostate cancer patients with worsening bone lesions that are not consistent with PSA trends.

Regarding the association between MM and prostate cancer, Kao et al. reported four cases of MM in 700 prostate cancer cases, and pointed out that a variety of cytokines produced by MM, such as insulin-like growth factor 1 (IGF-1), interleukin-6 (IL-6), stromal cell-derived factor 1 (SDF-1), and vascular endothelial growth factor (VEGF), as well as immunodeficiency states associated with MM, may contribute to prostate cancer development and progression and immunodeficiency states associated with MM may contribute to the development and progression of prostate cancer [9]. Differentiating prostate cancer patients presenting with bone lesions or orthopedic symptoms from those suffering from MM represents a significant clinical challenge.

Characteristic findings of MM include serum protein abnormalities typified by M protein production and lytic bone lesions resulting from osteoclast activation. However, distinguishing the latter from prostate cancer bone metastases through imaging alone is challenging. Huang et al.'s report also recommends performing bone marrow biopsy, in addition to bone scintigraphy and MRI, if possible [10].

MM is categorized based on the type of monoclonal heavy chain produced, including IgG, IgA, IgD, IgM, and, rarely, IgE. Additionally, 16% of MM cases are characterized by the secretion of FLC, either kappa or lambda, with a subset being non-secretory. Among these, IgE-type MM is particularly rare, accounting for only 0.1% of cases, and is associated with aggressive clinical behavior and a poor prognosis [4]. Diagnostic protocols typically involve the measurement of IgG, IgA, and IgM, often overlooking IgE and IgD. Consequently, the diagnosis of IgE myeloma tends to be delayed, contributing to its aggressive progression, including plasma cell leukemogenesis, and leading to an average prognosis of merely 16 months [11]. Accurate detection, quantification, and identification of the monoclonal component are essential, underscoring the importance of comprehensive protein assays [12].

The heightened risk of initial infections in patients with MM is a significant concern, attributed to compromised humoral and cellular immunity, decreased mobility, and diminished performance status factors intertwined with both the disease and its treatment modalities. It has been documented that infective complications account for the demise of up to 10% of patients within the first 60 days following diagnosis [13]. Renal impairment emerges as a common and serious complication of MM, presenting initially in 20-25% of patients and eventually affecting up to half of all patients at some stage of their illness. The presence of renal impairment in MM is an ominous prognostic factor [5]. Remarkably, renal insufficiency is reversible in about half of the affected patients, yet the rest continue to endure varying degrees of persistent renal dysfunction, with 2-12% necessitating renal replacement therapy. The primary mechanism of renal failure in MM is attributed to cast nephropathy or "myeloma kidney," resulting from damage to the renal tubules by FLCs [14]. Augustson et al. reported that among 299 patient deaths, 43 were directly related to renal failure within a 60-day period [13]. Knudsen et al. highlighted that infection and renal failure are principal contributors to early mortality in MM, emphasizing the critical need for renal failure prevention and reversal [15]. Early detection of both new and recurrent myeloma, as noted by Augustson et al., facilitates timely intervention and the avoidance of kidney damage [13].

In the presented patient, effective management of pyelonephritis, along with negating bone metastasis of neuroendocrine prostate cancer through bone marrow biopsy, and the relatively early diagnosis of IgE MM, were crucial in avoiding renal dysfunction and early mortality. Rapid progression of renal dysfunction, unexplained progressive anemia, back pain of unknown origin, hypercalcemia, and abnormal gamma globulin levels should raise suspicion of "myeloma kidney." Therefore, quantitative evaluation of M-protein, serum free light chain (SFLC) assay, and bone marrow biopsy are necessary to rapidly diagnose MM.

## Conclusions

Prostate cancer and MM should be noted as they may present with similar symptoms resulting from bone involvement. Poor prognostic types of MM also exist, such as IgE-mutant forms; moreover, acute renal failure may progress in MM, sometimes requiring dialysis treatment. In the management of prostate cancer, it is important to consider MM as a differential diagnosis when unexplained back pain, progressive anemia, signs of infection, and renal dysfunction develop despite the absence of significant changes in serum PSA levels. Prompt diagnosis of MM and therapeutic intervention are crucial to reduce worsening renal dysfunction and prevent early death.

## References

[1] Kundu S, Jha SB, Rivera AP (2022). Multiple myeloma and renal failure: mechanisms, diagnosis, and management. Cureus.

[2] Culp MB, Soerjomataram I, Efstathiou JA, Bray F, Jemal A (2020). Recent global patterns in prostate cancer incidence and mortality rates. Eur Urol.

[3] Adrianzen Herrera DA, Goldberg-Stein S, Sankin A, Sarungbam J, Sharma J, Gartrell BA (2018). Synchronous bone metastasis from multiple myeloma and prostate adenocarcinoma as initial presentation of coexistent malignancies. Front Oncol.

[4] Kehl N, Kilian M, Michel J (2022). IgE type multiple myeloma exhibits hypermutated phenotype and tumor reactive T cells. J Immunother Cancer.

[5] Knudsen LM, Hippe E, Hjorth M, Holmberg E, Westin J (1994). Renal function in newly diagnosed multiple myeloma - a demographic study of 1353 patients. The Nordic Myeloma Study Group. Eur J Haematol.

[6] Conteduca V, Oromendia C, Eng KW (2019). Clinical features of neuroendocrine prostate cancer. Eur J Cancer.

[7] Mai KT, Commons AS, Perkins DG, Yazdi HM, Collins JP (1996). Absence of serum prostate-specific antigen and loss of tissue immunoreactive prostatic markers in advanced prostatic adenocarcinoma after hormonal therapy: a report of two cases. Hum Pathol.

[8] Mahdy A, Patil R, Parajuli S (2019). Biochemical recurrence in prostate cancer and temporal association to bone metastasis. Am J Case Rep.

[9] Kao J, Jani AB, Vijayakumar S (2004). Is there an association between multiple myeloma and prostate cancer?. Med Hypotheses.

[10] Huang E, Teh BS, Saleem A, Butler EB (2002). Recurrence of prostate adenocarcinoma presenting with multiple myeloma simulating skeletal metastases of prostate adenocarcinoma. Urology.

[11] Macro M, André I, Comby E (1999). IgE multiple myeloma. Leuk Lymphoma.

[12] Nafría Jiménez B, Oliveros Conejero R (2022). IgE multiple myeloma: detection and follow-up. Adv Lab Med.

[13] Augustson BM, Begum G, Dunn JA (2005). Early mortality after diagnosis of multiple myeloma: analysis of patients entered onto the United kingdom Medical Research Council trials between 1980 and 2002 - Medical Research Council Adult Leukaemia Working Party. J Clin Oncol.

[14] Clark AD, Shetty A, Soutar R (1999). Renal failure and multiple myeloma: pathogenesis and treatment of renal failure and management of underlying myeloma. Blood Rev.

[15] Knudsen LM, Hjorth M, Hippe E (2000). Renal failure in multiple myeloma: reversibility and impact on the prognosis. Nordic Myeloma Study Group. Eur J Haematol.

